# Contrasting Responses of a Native Palm and an Invasive Vine to Flooding Stress: Implications for Orchard Regeneration in Caatinga Ecosystems

**DOI:** 10.3390/plants15060954

**Published:** 2026-03-20

**Authors:** Wiliana Júlia Ferreira de Medeiros, Claudivan Feitosa de Lacerda, Bruno Gabriel Monteiro da Costa Bezerra, Jonnathan Richeds da Silva Sales, Francisco Gleyson da Silveira Alves, Elayne Cristina Gadelha Vasconcelos, Magno José Duarte Cândido, Paula Ingrid Maia Machado, Oriel Herrera Bonilla, Isabel Cristina da Silva Araújo, Carlos Henrique Carvalho de Sousa, Antonio Marcos Esmeraldo Bezerra

**Affiliations:** 1 Center of Agrarian Sciences, Federal University of Ceará (UFC), Av. Mr. Hull 2877, Fortaleza 60356-001, CE, Brazil; juliamedeirosagro@gmail.com (W.J.F.d.M.); brunog768@gmail.com (B.G.M.d.C.B.); jonnathanagro@gmail.com (J.R.d.S.S.); gleyson@ufc.br (F.G.d.S.A.); elaynegadelha@gmail.com (E.C.G.V.); magno@ufc.br (M.J.D.C.); paulaingrid.mm@gmail.com (P.I.M.M.); isabelaraujo@ufc.br (I.C.d.S.A.); esmeraldo@ufc.br (A.M.E.B.); 2 Center of Health Sciences, State University of Ceará (UECE), Av. Dr. Silas Munguba 1700, Fortaleza 60740-903, CE, Brazil; oriel.herrera@uece.br; 3 Ieducare Faculty (FIED), R. Conselheiro João Lourenço 406, Tianguá 62320-000, CE, Brazil; carloshenrique@fied.edu.br

**Keywords:** caatinga ecosystem, carnauba palm, biological invasion, soil flooding

## Abstract

The carnauba palm trees in the Caatinga ecosystem, in Northeast Brazil, have been impacted by invasive species, particularly in areas subject to flooding. This study aimed to evaluate morphological, physiological, and nutritional responses of *Copernicia prunifera* (native) and *Cryptostegia madagascariensis* (invasive) seedlings exposed to flooding stress. The experiment was conducted in a randomized complete block design, with a split-plot arrangement and five replicates. The treatments were formed by two species and five periods of flood stress (0, 8, 12, 16, and 20 days). Flooding significantly reduced shoot dry mass in both species; however, the reduction was more pronounced in the invasive species (27%) compared to the native palm (20%). The invasive species showed strong use of resources, with higher values for leaf mineral nutrient, net photosynthesis, growth rate, and leaf area, regardless of the water regime. Under flooding, the invasive species produced adventitious roots, and the net photosynthetic rate was less impacted, despite greater sodium accumulation in the leaves. The results indicate that the characteristics of *C. prunifera*, such as slow growth rate, low specific leaf area, and morphological adaptations of the root system, may ensure greater stability in net carbon assimilation in the whole plant under flooding. However, the rapid growth and high absorption of soil resources of *C. madagascariensis* pose a significant threat to the establishment of *C. prunifera* seedlings, directly jeopardizing the long-term renewal of carnauba palm groves in the Caatinga ecosystem.

## 1. Introduction

Native tree species can play a key role in addressing the consequences of climate changes. As products of a long process of natural selection, these species possess genes resistant to abiotic constraints, such as rising air temperature, soil salinity, drought, and flooding [1]. Most native Brazilian species exhibit different adaptive strategies that enhance their use in various productive sectors, as well as in the reclamation of degraded areas [2].

The Caatinga ecosystem, a large geographical area (850,000 km^2^) located in Northeast Brazil, comprises different types of vegetation under a sub-humid and semi-arid tropical climate [3,4,5], including extensive areas of the *Copernicia prunifera* (Carnauba palm). The populations of this palm play an important role in ecological balance and may be suitable for programs to recover degraded areas [6]. Carnauba palms also constitute a source of income for the population, through wax extraction, wood utilization, and the manufacture of ornamental products [7].

Plants of *C. prunifera* grow and disperse along flooded and waterlogged areas, contributing to the maintenance of water bodies and preventing problems such as river siltation and soil erosion [2,7]. The periodic occurrence of flooding in carnauba palm groves indicates that this species has adaptations to withstand anoxic conditions [8]. However, these areas have been invaded by the exotic species *Cryptostegia madagascariensis*, a woody vine native to Madagascar that forms dense thickets due to its vigorous climbing growth and high biomass production, establishing dense populations particularly in areas near rivers and lagoons. The climbing branches overtop native vegetation, competing for soil resources and blocking light penetration and causing the death of affected individuals through shading [9,10,11,12,13].

Understanding the ecophysiological changes triggered in plants by different abiotic constraints is fundamental for the development of mitigation strategies. For example, long periods of flooding cause drastic reduction in the availability of oxygen in the soil, promoting reductions in leaf gas exchange and chlorophyll content [14]. In response to this condition, some plants may activate tolerance mechanisms, such as the formation of aerenchyma and adventitious roots [15,16,17,18,19]. Although flooded and waterlogged are common habitats for native (*C. prunifera*) and invasive species (*Cryptostegia madagascariensis*), little is known about the comparative ecophysiology and survival strategies during the seedling stage, a critical phase that determines successful establishment and long-term population dynamics under soil water excess.

According to [13], the competitive interaction between adult plants of *C. prunifera* and *C. madagascariensis* is mainly defined by the species’ adaptability to abiotic soil constraints, including salinity, water deficit, and water excess. Infestation by *C. madagascariensis* is more intense under waterlogged conditions and in salt-affected soils, since the ecophysiological responses of this invasive species are not severely affected, even with high sodium accumulation in its leaves. This competitive capacity of the invasive plant may be decisive in the renewal of carnauba palm groves, as it is hypothesized that the effects of this competition may be even more drastic in the establishment of seedling of *C. prunifera*, especially in areas subject to flooding or waterlogging for long periods. Given this context, the objective of this study was to evaluate the physiological, nutritional, and morphological responses in young plants of *C. prunifera* and *C. madagascariensis* exposed to periods of excess water.

## 2. Results

### 2.1. Leaf Gas Exchange, Chlorophyll Fluorescence, and Relative Chlorophyll Index

Leaf gas exchange was influenced by the interaction between flooding and sampling time and between species and sampling time (*p* < 0.01). Chlorophyll fluorescence and the SPAD index responded to the effects of the triple interaction between species x flooding x evaluation time (*p* < 0.05).

**Figure 1:**
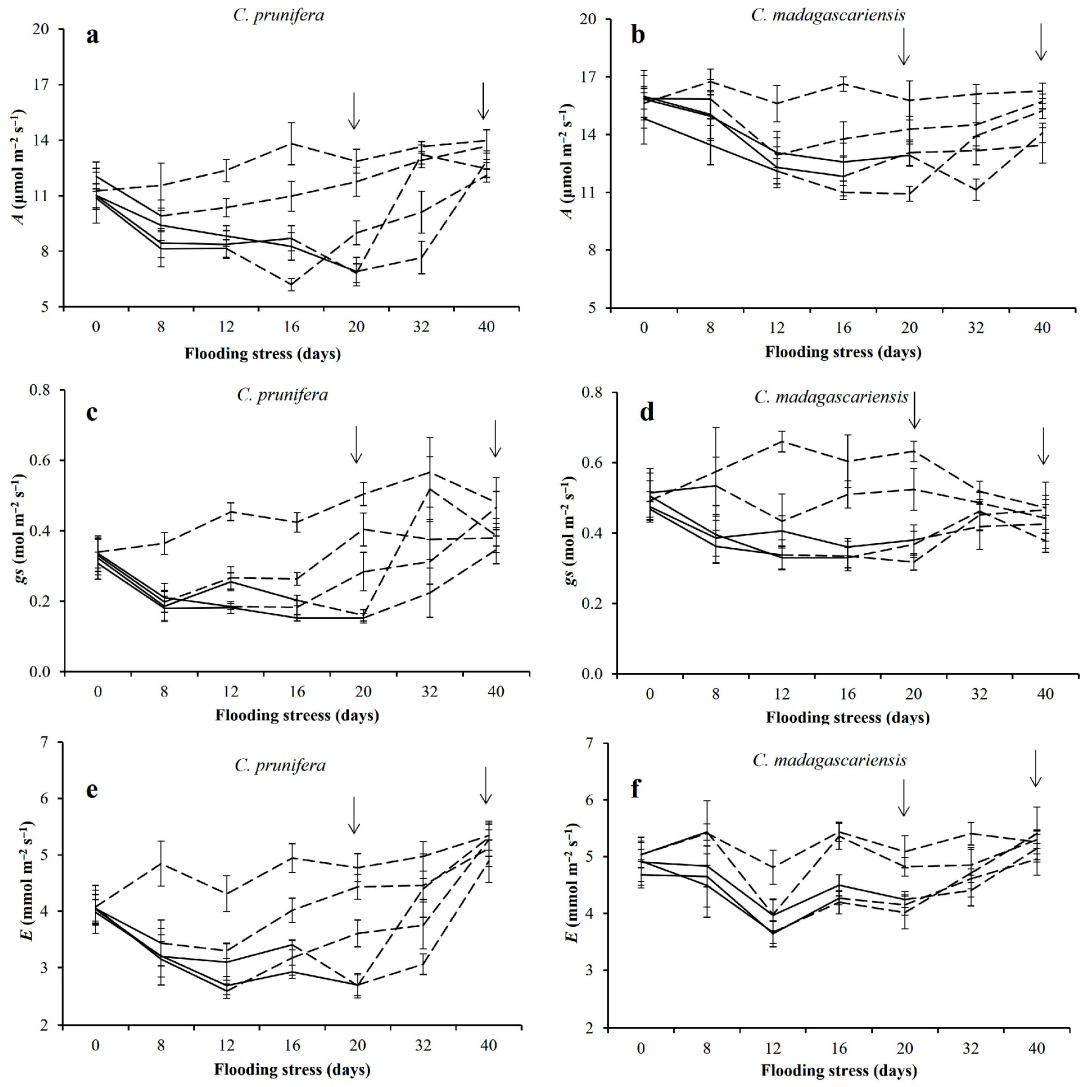
Gas exchange in mature leaves of *C. prunifera* (**a**,**c**,**e**) and *C. madagascariensis* (**b**,**d**,**f**) seedlings under different flooding periods. Net photosynthetic rate-*A* (**a**,**b**), stomatal conductance-*gs* (**c**,**d**), and transpiration rate-*E* (**e**,**f**). Solid lines (—) indicate stress periods (8, 12, 16, and 20 days) and dashed lines (---) indicate the absence or suspension of stress. Arrows indicate the end of the longest flooding period (20 days) and recovery (40 days).

Leaf gas exchange data indicates that plants of both species survive relatively long periods of flooding (Figure 1), showing inhibition in *A*, *gs*, and *E* with the imposition of stress and a subsequent increase in these responses during recovery periods. Reductions of 14% and 5% in the net photosynthetic rate (*A*) were observed on the 8th day of stress for *C. prunifera* and *C. madagascariensis*, respectively. With the increase in the stress period (20 days of flooding), reductions reached 46% and 18% in the net photosynthetic rates of *C. prunifera* and *C. madagascariensis*, respectively (Figure 1a,b). Reductions in *gs* and *E* values were also observed in *C. prunifera* plants, reaching 70% and 44% at the end of the stress period, respectively (Figure 1c,e). For *C. madagascariensis*, these reductions were 40% and 17% for *gs* and *E* (Figure 1d,f), respectively, when compared to the control treatment (without flooding). Twenty days after the end of flooding stress (Figure 1), photosynthesis rates reached values equal to or greater than 85%, compared to control plants (without flooding), even in those plants that were subjected to long periods of flooding.

**Figure 2:**
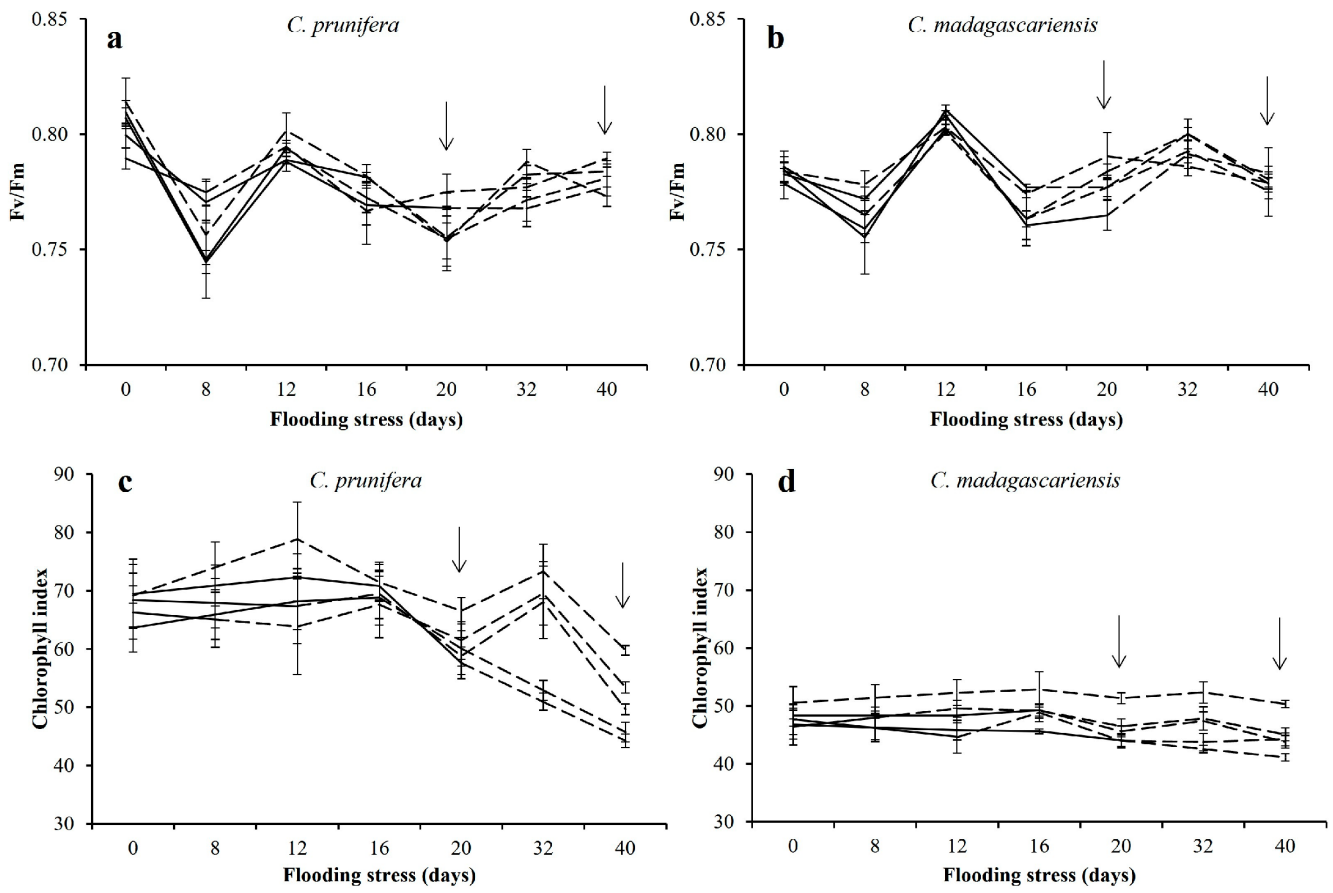
Chlorophyll fluorescence and relative chlorophyll index (RIC) in mature leaves of *C. prunifera* (**a**,**c**) and *C. madagascariensis* (**b**,**d**) seedlings under different flooding periods. The Fv/Fm ratio (**a**,**b**) and the relative chlorophyll index (**c**,**d**). Solid lines (—) indicate stress periods (8, 12, 16, and 20 days) and dashed lines (---) indicate the absence or suspension of stress. Arrows indicate the end of the longest flooding period (20 days) and recovery (40 days).

The Fv/Fm ratio showed variations over time but not apparently related to the length of periods of flood stress (Figure 2). In relative terms, decreases of 3% were observed in the Fv/Fm ratio of *C. madagascariensis*, and no decreases were observed for *C. prunifera*, after 20 days of flooding (Figure 2a,b).

Plants of *C. prunifera* and *C. madagascariensis* that were flooded for 20 days showed reductions in relative chlorophyll content of 13% and 14%, respectively (Figure 2c,d). However, a downward trend in this index was observed in *C. prunifera* during the recovery period, apparently unrelated to flooding stress. For *C. madagascariensis*, the index of chlorophyll at the end of the stress periods were lower in plants that received flooding, regardless of the duration of the stress.

### 2.2. Morphological Responses and Biomass Production

**Figure 3:**
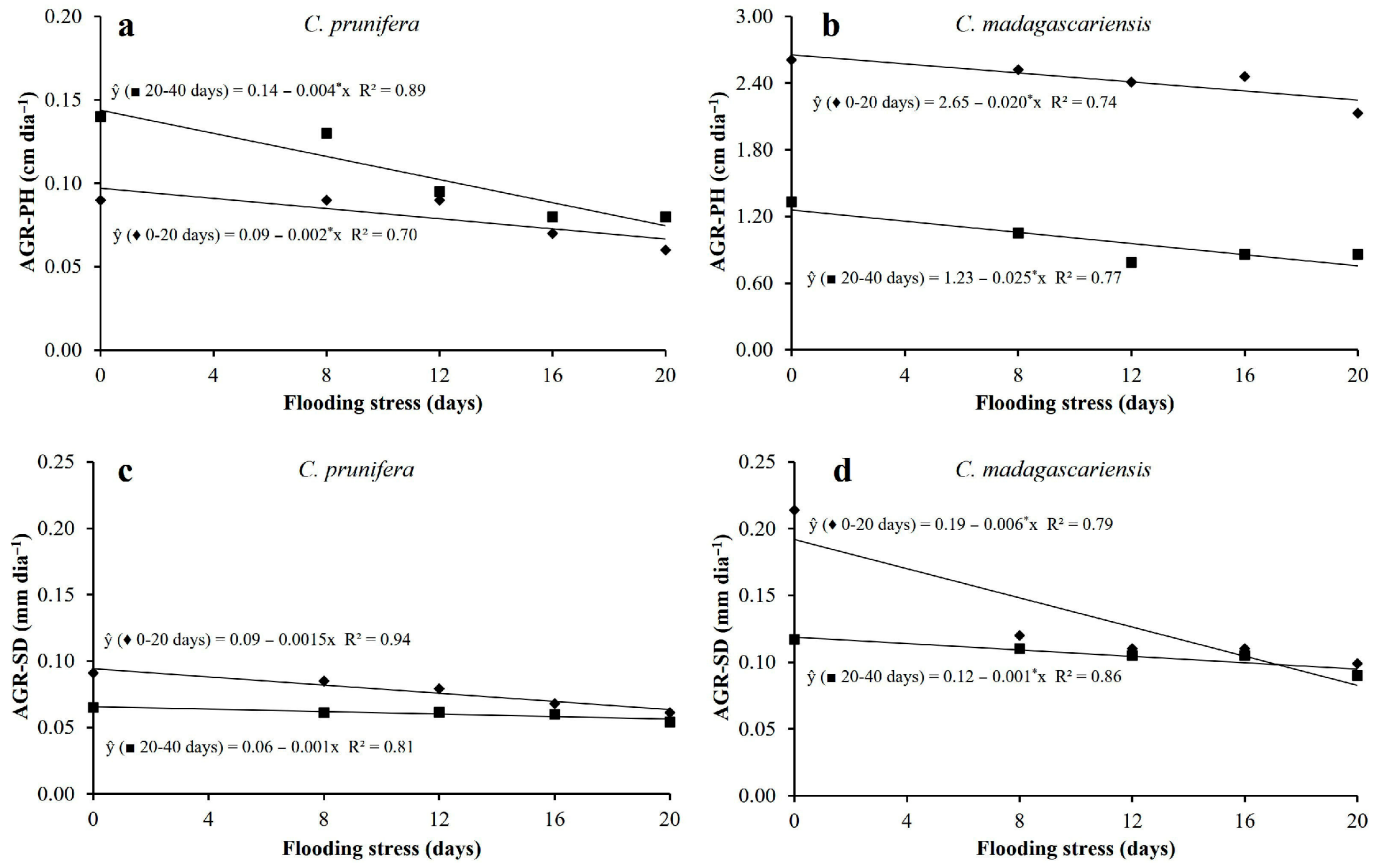
Absolute growth rates of *C. prunifera* (**a**,**c**) and *C. madagascariensis* (**b**,**d**) seedlings under different flooding periods. Absolute growth rate for plant height (AGR–PH) is shown in (**a**,**b**), and absolute growth rate for stem diameter (AGR–SD) is shown in (**c**,**d**). * —Significant at *p* ≤ 0.05, respectively, by the F-test.

Both species showed inhibition in absolute growth rates for plant height and stem diameter when subjected to flooding stress. In general, growth rates were lower in *C. prunifera* compared to *C. madagascariensis* (Figure 3).

For *C. prunifera*, reductions of 33% and 43% were observed in plant height growth rate and 33% and 17% in absolute stem diameter growth rate between 0–20 and 20–40 days, respectively, when comparing the control treatment with plants that grew for 20 days under stress (Figure 3a,c). *C. madagascariensis* showed decreases in height growth rates of 18% and 35% when comparing the extreme treatments (control and 20 days of flooding). For the absolute stem diameter growth rate, these reductions reached 53% and 23%, respectively (Figure 3b,d).

**Figure 4:**
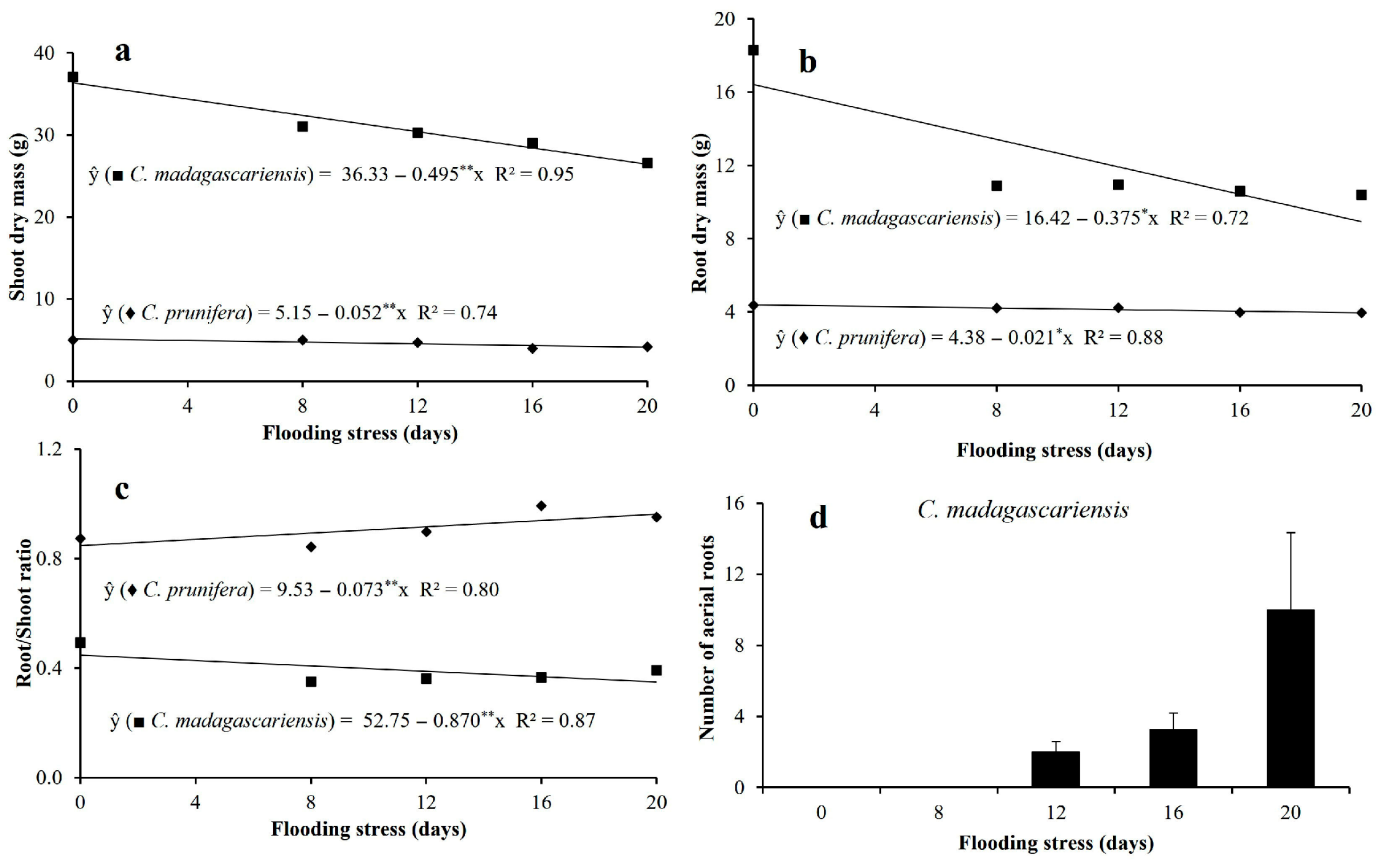
Biomass production and morphological traits of *C. prunifera* and *C. madagascariensis* seedlings under different flooding periods. Shoot dry mass-SDM (**a**), root dry mass-RDM (**b**), root/shoot ratio (**c**), and number of adventitious roots (**d**). ** and * —Significant at *p* ≤ 0.01 and *p* ≤ 0.05, respectively, by the F-test.

For shoot dry mass and root dry mass, *C. madagascariensis* plants showed more pronounced reductions, of 0.49 and 0.38 g day^−1^, respectively (Figure 4). For *C. prunifera*, these reduction rates were 0.05 and 0.02 g day^−1^, respectively. In relative terms, maximum reductions in shoot dry mass and root dry mass of 20.2 and 9.6% were observed for *C. prunifera* (Figure 4a) and 27.3 and 45.7% for *C. madagascariensis*, respectively, when comparing the treatments that were subjected to 0 (control) and 20 days of flooding (Figure 4b).

The different responses of shoots and root growth to flooding resulted in a 23.8% increase in the root/shoot ratio of the native species and a 22.2% reduction in the invasive species (Figure 4c). However, the invasive species showed adventitious root formation after the 12th day of stress, with significant production at 20 days of stress (Figure 4d).

The distribution of roots in the soil layers differed between the two species, depending on the time of exposure to stress. In the control treatment there was a relatively uniform distribution of roots within the pot for both species (Table 1). For *C. prunifera* this trend remained until 12 days of flooding, with the deepest layer containing about 31% of the root mass. However, after 16 and 20 days of flooding, the percentage of roots at the greatest depth dropped to 15.1 and 18.4%, respectively. For the species *C. madagascariensis*, root distribution was uniform only in control treatment, with similar values in the surface layer (0 to 8 cm) and the deeper layer (16 to 24 cm). However, for the treatments with 8, 12, 16, and 20 days of flooding, the percentages of root biomass in the surface layer reached 65.2, 65.3, 67.6, and 78%, respectively. The appearance of adventitious roots only in the invasive species also confirms the differences in response to flooding compared to the native species, as shown in Figure 4d.

**Figure 5:**
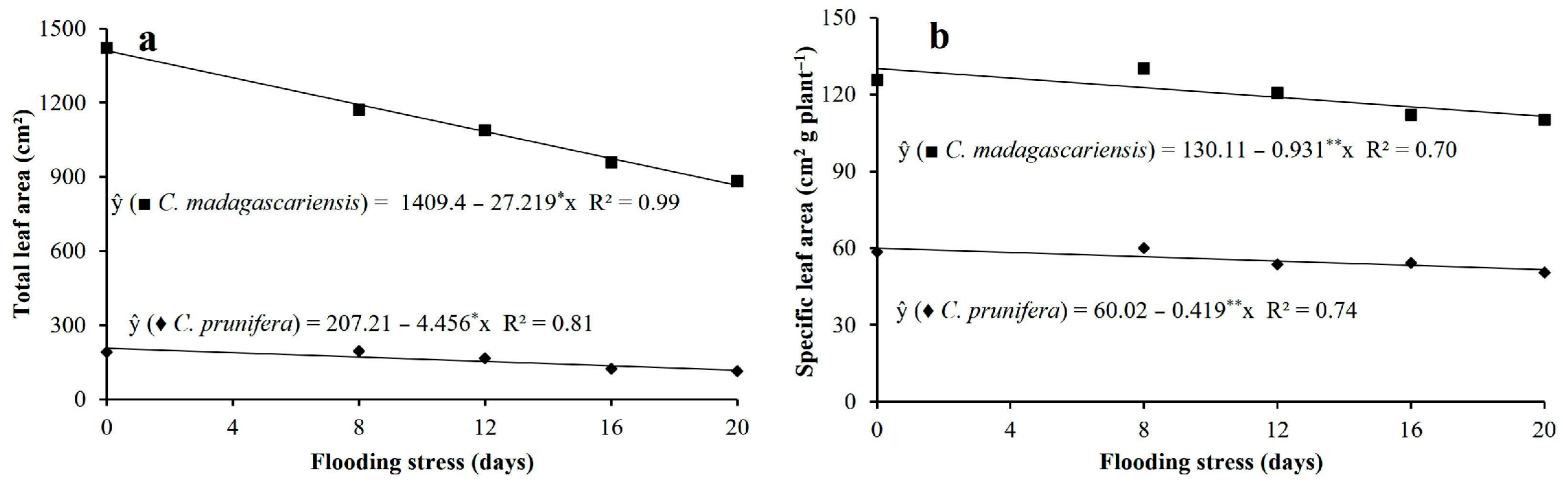
Total leaf area (**a**) and specific leaf area (**b**) of *C. prunifera* and *C. madagascariensis* seedlings under different flooding periods. ** and * —Significant at *p* ≤ 0.01 and *p* ≤ 0.05, respectively, by the F-test.

In general, flooding stress promoted linear reductions of 43.0 and 38.0% in total leaf area (Figure 5a) and 14.0 and 14.3% in specific leaf area (Figure 5b) in the species *C. prunifera* and *C. madagascariensis*, respectively. However, leaf area and specific leaf area are significantly larger in the invasive species, regardless of the flooding exposure time.

### 2.3. Mineral Nutrition

**Figure 6:**
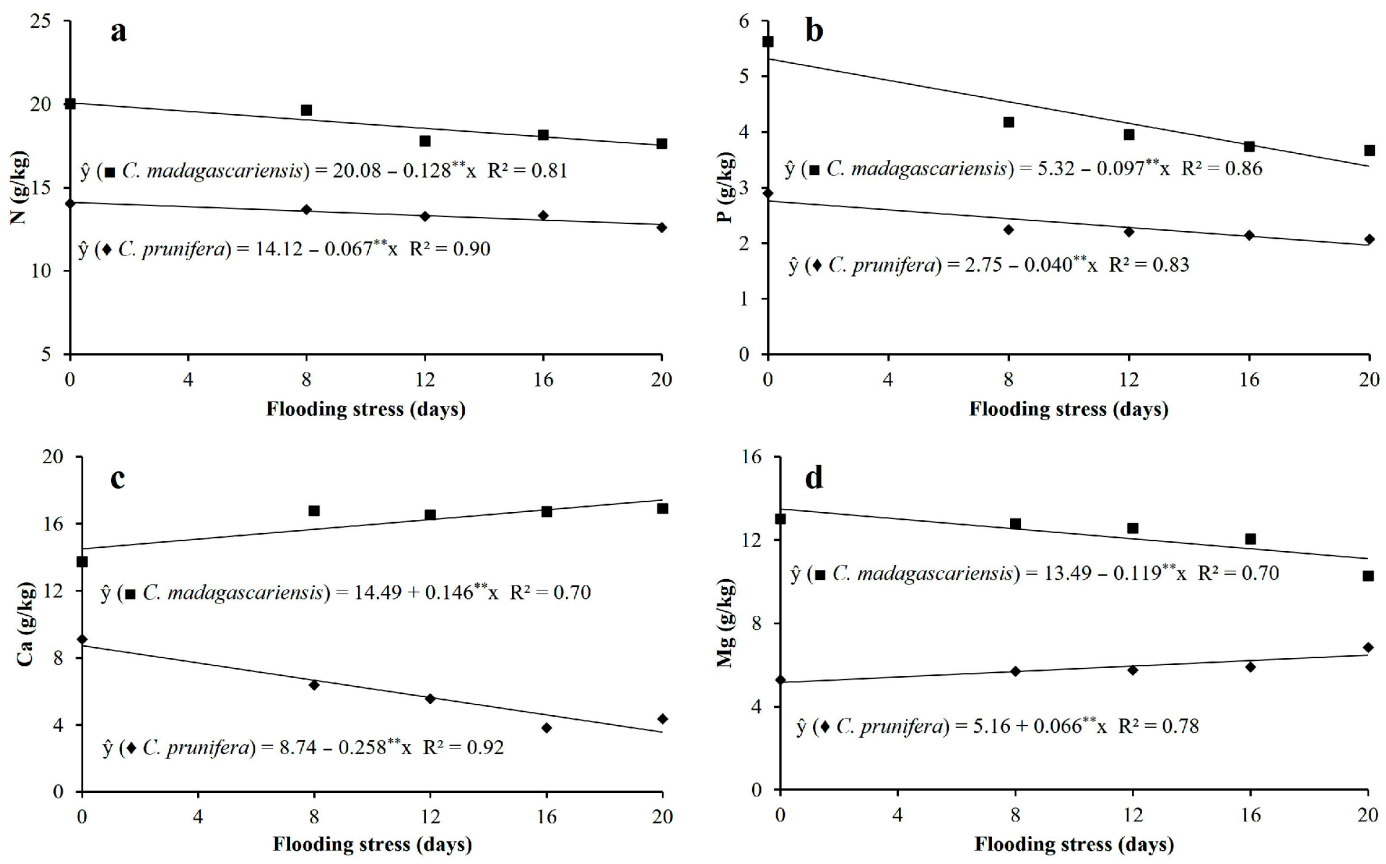
Leaf concentration of nitrogen-N (**a**), phosphorus-P (**b**), calcium-Ca (**c**) and magnesium-Mg (**d**) in mature leaves of *C. prunifera* and *C. madagascariensis* seedlings under different flooding periods. ** and * —Significant at *p* ≤ 0.01 and *p* ≤ 0.05, respectively, by the F-test.

The concentrations of N, P, Ca, and Mg were higher in the leaves of *C. madagascariensis* than in the native species, regardless of the flooding cycles. Nitrogen and phosphorus levels showed linear decreases in the leaves of plants of both species with the imposition of flooding stress (Figure 6a,b). In relative terms, a similar trend was observed for both species, with maximum reductions in N and P of 10 and 29% for *C. prunifera* and 12 and 34% for *C. madagascariensis*, respectively, comparing the treatments that were subjected to 0 and 20 days of flooding.

The Ca and Mg concentrations showed opposite response for *C. prunifera* and *C. madagascariensis* when the plants were subjected to flooding periods. For *C. prunifera*, reductions of up to 52% in calcium and an increase of 23% in magnesium were observed. For *C. madagascariensis*, an increase of 29% in calcium and a reduction of 21% in leaf magnesium concentrations were observed when the plants were subjected to 20 days of stress compared to the control treatment (Figure 6c,d).

**Figure 7:**
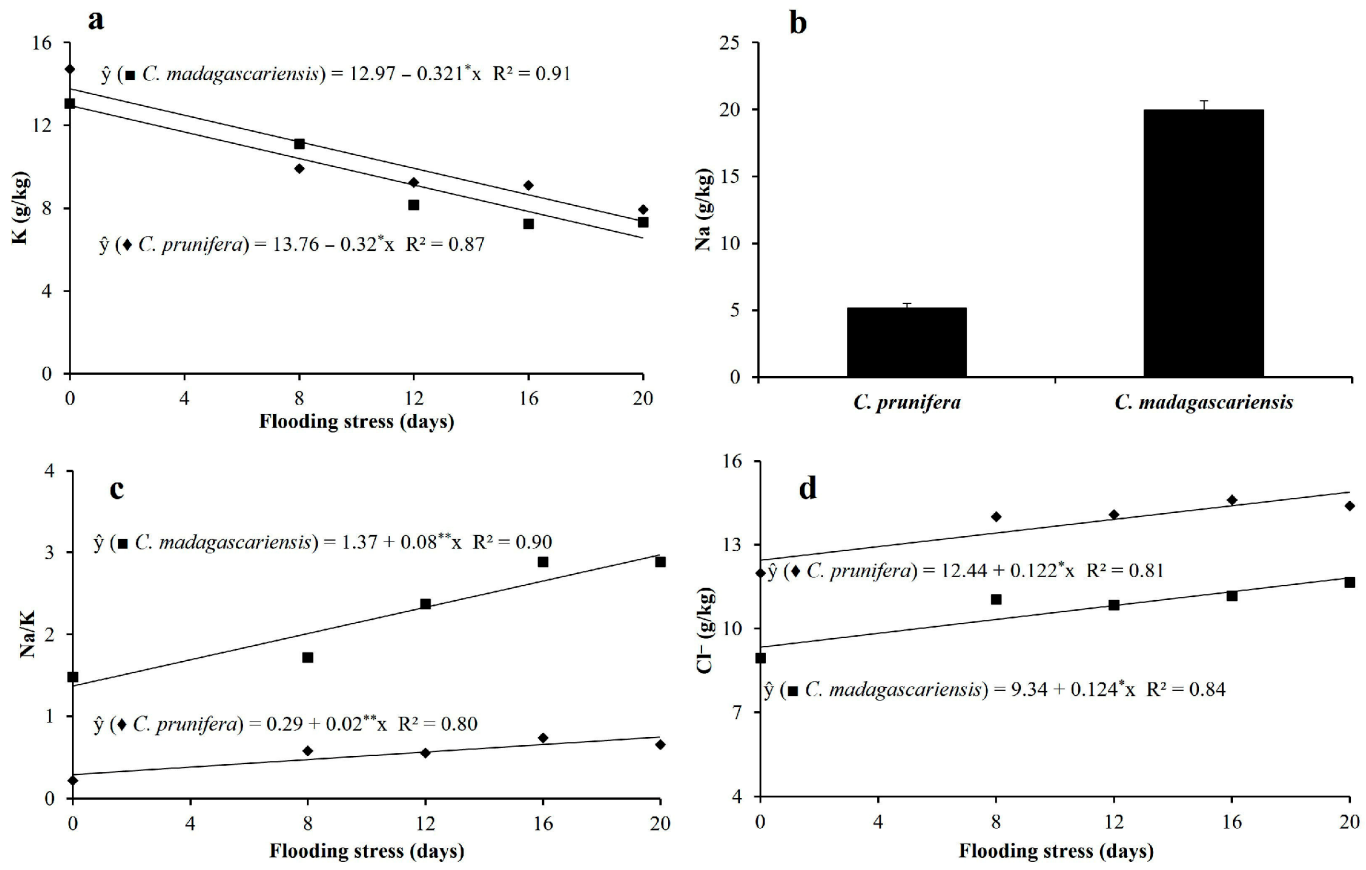
Leaf concentration of potassium-K (**a**) sodium-Na (**b**), sodium/potassium ratio-Na/K (**c**) and chloride-Cl (**d**) in mature leaves of *C. prunifera* and *C. madagascariensis* seedlings under different flooding periods. ** and * —Significant at *p* ≤ 0.01 and *p* ≤ 0.05, respectively, by the F-test.

Potassium concentrations were similar and decreased linearly in both species with flooding time (Figure 7a). Otherwise, sodium concentrations were not impacted by flooding stress and were approximately 4 times higher in the leaves of *C. madagascariensis* compared to *C. prunifera* (Figure 7b). The Na/K ratio increased with the number of days of flooding stress but was always below 1.0 in the native species (Figure 7c). In the invasive species, the Na/K ratio ranged from 1.5 to 3.0. In relative terms, increases of 20 and 30% were observed in the chloride concentrations of *C. prunifera* and *C. madagascariensis*, respectively, when subjected to 20 days of flooding stress, compared to the control treatment (Figure 7d).

## 3. Discussion

Oxygen deficiency and low soil redox potential, induced by flooding and waterlogging, adversely affect plant growth by limiting carbon assimilation, nutrient uptake, and root respiratory metabolism [20,21]. Many tree species, however, can survive prolonged flooding [16,22], including populations of carnauba palms in the Caatinga ecosystem [8,23,24]. Despite these adaptations, native populations have been increasingly affected by competition with the invasive species *C. madagascariensis*, particularly under excess soil water [13]. The impacts of this abiotic constraint during seedling stage of native and invasive species can be crucial to explain the current replacement dynamics observed in these areas.

We evaluated the responses of young plants of *C. prunifera* and *C. madagascariensis*, and the species responded differently to flooding at physiological, nutritional, and morphological levels. In general, the invasive species exhibited faster growth and greater mineral nutrient accumulation regardless of water regime, whereas the native species showed slower but more stable growth under excess soil water. These results reveal two contrasting functional strategies: a resource acquisitive strategy in *C. madagascariensis* and a resource conservative strategy in *C. prunifera* under flooding conditions.

Leaf gas exchange data showed that *C. prunifera* seedlings were more sensitive to flooding than *C. madagascariensis*, corroborating observations in adult plants [13]. The invasive species maintained higher net photosynthesis rates under both control and flooded conditions. This sustained values of momentaneous carbon assimilation under hypoxic stress suggests greater metabolic flexibility and stomatal regulation efficiency [12,13]. Both species demonstrated recovery of leaf gas exchange after drainage, indicating reversible functional limitation within the tested flooding period.

**Figure 8:**
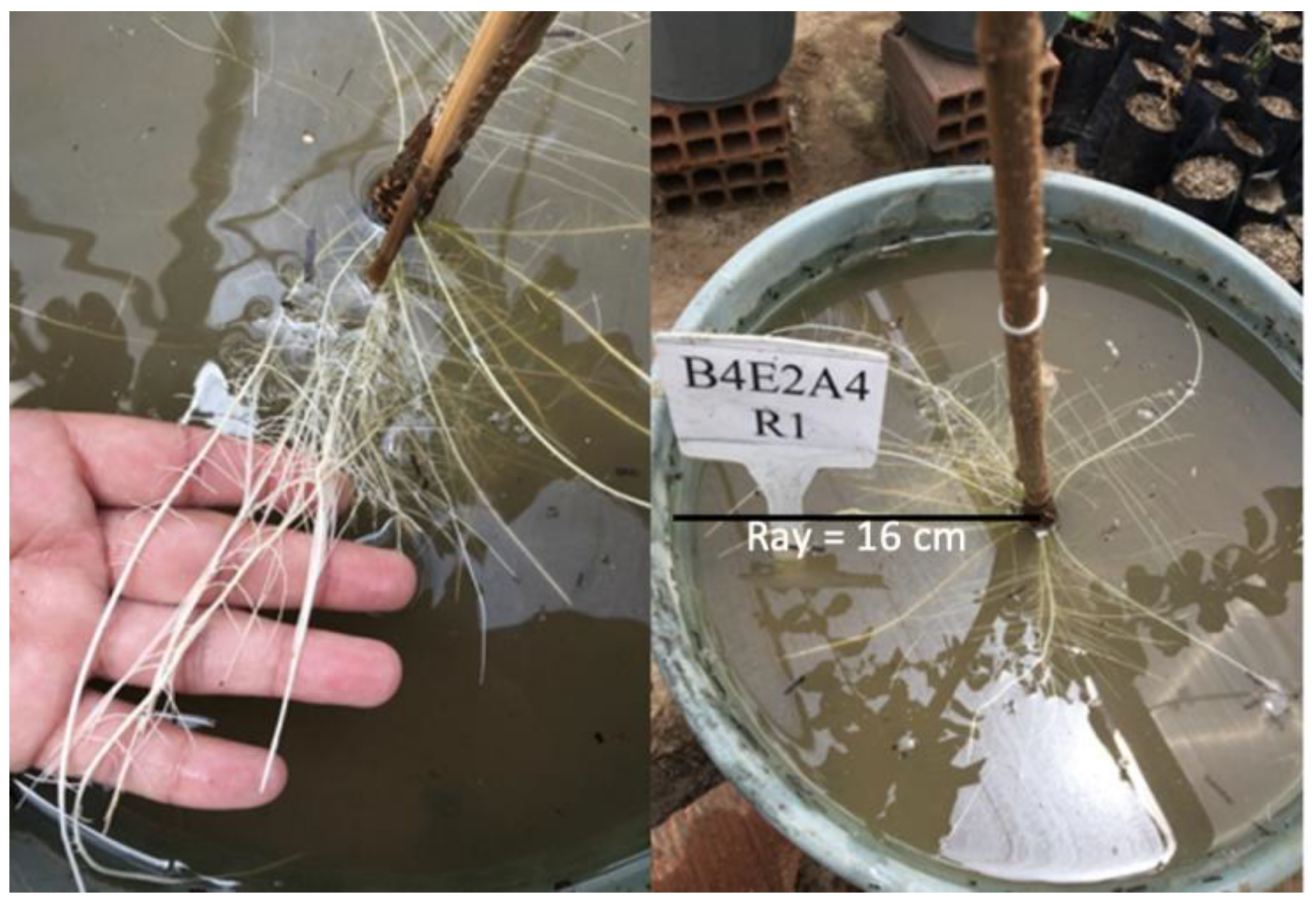
Adventitious roots of *C. madagascariensis* subjected to a 20-day flooding period.

Flooding effects on growth were more pronounced in *C. madagascariensis* under prolonged exposure. Periods longer than 12 days induced metabolite reallocation toward adventitious root formation (Figure 8). This response indicates a carbon trade-off, prioritizing structural adaptations that enhance internal aeration over vertical growth. The high density of lenticels favors adventitious root development, improving oxygen diffusion under hypoxia [25,26]. Root system of the invasive species became concentrated in superficial soil layers, a common adaptive response in flooded environments [27,28], although this strategy may increase dependence on short-term hydrological stability (Table 1).

In contrast, *C. prunifera* maintained a higher root/shoot ratio and more uniform root distribution in the soil profile. This structural stability, combined with controlled stomatal conductance, suggests a conservative hydraulic strategy that minimizes metabolic imbalance under prolonged stress. The maintenance of leaf integrity and moderate reductions in growth reinforce its tolerance to extended flooding [13]. According to [8], the high porosity of the root system is a characteristic that favors the development of *C. prunifera* under flooding. This species also maintained its leaves intact, controlling stomatal opening and water loss by transpiration.

Thus, the distinct strategies presented by *C. madagascariensis* and *C. prunifera* demonstrate that responses to flooding depend on specific morphophysiological adjustments. This adaptive variability reinforces that plant responses to flooding are complex and vary between species and duration of stress [14,29]. *C. prunifera* exhibits lower growth rates than *C. madagascariensis*, and this adaptation may be efficient when subjected to prolonged soil water excess, resulting in smaller reductions in leaf area and biomass accumulation. The low specific leaf area in *C. prunifera*, combined with strong stomatal control, contributes to lower net photosynthesis rates per unit of leaf area, but ensures greater stability in whole-plant growth under flooding. The maintenance of leaf area, associated with better root distribution in the soil, indicates the ability of *C. prunifera* to survive for longer flooding periods than those tested in this study, which are common in carnauba palm groves during the rainy season in the Caatinga ecosystem.

The restriction to leaf development is detrimental to plants under stress, since leaf area is directly linked to the plant’s photosynthetic rate, and when reduced, it severely affects the production of photoassimilates [30]. Our results showed that flooding caused similar impacts on both species in terms of leaf area reduction. However, the smaller specific leaf area and greater leaf longevity may result in more uniform photosynthetic rates in the native species, differentiating it from the invasive species, which has short-lived leaves. This distinction in leaf characteristics suggests that, despite experiencing similar reductions in leaf area, the species adopt distinct functional strategies to mitigate the effects of flooding, which may influence their stress tolerance and recovery capacity. These functional differences are also related to the fact that plant species have their own leaf architectures and morphologies, as well as anatomical, physiological, and biochemical characteristics that modulate their responses to stress [13]. Therefore, the contrasting strategies observed between the native and invasive species reflect not only immediate adjustments to flooding, but also intrinsically determined genetic attributes that influence their tolerance to excess water in the soil [31].

Our research found that soil flooding restricts the absorption of N, K, and P and increases Cl^−^ accumulation in both species. responses also observed in other studies [13,32,33]. However, the consistently higher foliar nutrient concentrations observed in *C. madagascariensis* indicate intrinsic differences in nutrient acquisition efficiency between the species. The invasive species maintained higher photosynthetic rates, which likely ensured a greater supply of photoassimilates to sustain energy dependent nutrient uptake under hypoxic conditions. In addition, the root adjustments in invasive species under flooding may have enhanced soil exploration in the top soil, improving access to available nutrients.

Excess water in the root zone inhibits aerobic respiration with a simultaneous inhibition in mitochondrial ATP synthesis, reducing plant energy levels and, consequently, the capacity for nutrient absorption by the roots and transport to the shoots [34,35]. Flooding stress can also inhibit the activity of several transporters involved in nutrient absorption and transport [28].

The most important aspect revealed in our study was the difference between seedlings of the species in terms of nutrient accumulation in leaf tissues. The invasive species showed much higher foliar concentrations of minerals (N, P, K, Ca, Mg, and Na) than the native species, regardless of the water regime. This response, associated with a larger leaf area and greater biomass accumulation, indicates a high capacity of *C. madagascariensis* to extract nutrients from the soil. The high accumulation of Na and the high Na/K ratio in the leaves of this species stand out, reinforcing its aggressive character and its ability to adapt to the carnauba palm habitat. This ability to adjust the ionic balance, even under adverse conditions, contributes to its success in colonizing and dominating wetlands, competing with native species. The high sodium content and Na/K ratio (ranged from 1.5 to 3.0) indicate that *C. madagascariensis* exhibits halophytic behavior. These results indicate the high efficiency of the invasive species in sodium compartmentalization, as well as its ability to maintain cellular osmotic and ionic homeostasis [13,27,36].

Overall, the predominance of *C. madagascariensis* under flooded conditions appears to result from the integration of three key functional attributes: sustained carbon assimilation rate under hypoxic stress; high efficiency for absorption of essential nutrients from the soil; and effective ionic regulation, particularly Na^+^ compartmentalization. Together, these traits characterize a highly acquisitive strategy that maximizes growth and resource capture during seedling establishment. In contrast, *C. prunifera* exhibits a conservative strategy based on structural stability, slow growth, and greater tolerance to prolonged flooding. Although this strategy enhances survival, it may be insufficient to counterbalance the aggressive resource capture and expansion capacity of the invasive species under current environmental conditions.

## 4. Materials and Methods

### 4.1. Location and Characterization of the Area

**Figure 9:**
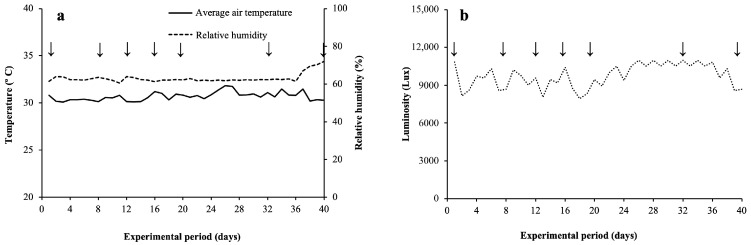
Average data for air temperature, relative air humidity (**a**), and luminosity (**b**) during the experimental period. Arrows indicate the beginning and end of each flooding period (0, 8, 12, 16, and 20 days) and recovery period (32 and 40 days).

The study was conducted in a greenhouse located in the city of Fortaleza (03°45′ S; 38°33′ W, 19 m altitude), Ceará, Brazil. The air temperature, relative humidity, and luminosity data obtained during the study with an Onset brand data logger, model Hobo^®^(Onset Computer Corporation, Bourne, MA, USA), are presented in Figure 9.

### 4.2. Experimental Design and Treatments

The experiment was conducted under a randomized block design, in a split-plot arrangement with five replications. The treatments in the main plots consisted of two species (*C. prunifera* and *C. madagascariensis*), simulating the establishment of seedlings in the field. The treatments in the subplots consisted of five periods of flooding stress (0, 8, 12, 16, and 20 days). Each experimental unit consisted of two pots, with one plant per pot. After each flooding period, the excess water was drained from the pots, maintaining the soil at field capacity. For the variables where data were collected over time, the statistical design was arranged in sub-subplots, adding the sampling time as a source of variation.

### 4.3. Growing Conditions

Seedlings of *C. prunifera* and *C. madagascariensis* were produced from seeds collected from populations of these species at Fazenda Raposa (3°50′ S; 38°38′ W) located in the municipality of Maracanaú, Ceará, Brazil. The seedlings were produced in polyethylene bags measuring 14 × 28 cm. The substrate used was composed of sand and organic compost in a 3:1 ratio. *C. prunifera* seedlings were transplanted at five months of age and *C. madagascariensis* seedlings at two months of age. The difference in chronological age reflects intrinsic differences in growth rate between the species. Transplantation was performed when seedlings of both species reached a comparable developmental stage, characterized by similar plant height, number of fully expanded leaves, and established root systems. Therefore, standardization was based on morphological and physiological equivalence rather than chronological age alone, ensuring experimental comparability.

Pots with a volumetric capacity of 20 L were used, with a hole at the bottom to promote the drainage of any excess water at the end of each flooding period. Initially, a layer of gravel (3 cm thick) was placed at the bottom of the pot to facilitate the free drainage of excess water. This gravel layer was covered with a non-woven geotextile membrane to prevent possible soil loss. The soil used as a substrate for cultivation came from the municipality of Caucaia, Ceará, Brazil, Fazenda Várzea dos Buracos (03°41′ S; 38°53′ W), and is classified as a Haplic Planosol. This area has populations of adult carnauba palms infested by *C. madagascariensis*. Approximately five tons of soil were collected at depths of 0–20 and 20–40 cm. Composite samples were collected for characterization of the soil’s chemical attributes, and the results are described in [13].

After transplanting, the seedlings were irrigated every other day, maintaining the soil at field capacity to ensure initial establishment. The water used for irrigation came from a groundwater well with an electrical conductivity of 1.0 dS m^−1^. The flooding treatments were initiated 30 days after transplanting (DAT), maintaining a water level of approximately 3 cm above the soil surface, with evaporation losses being replenished as needed. After the flooding cycles, the pots were drained and the excess water collected and returned to the pots to avoid losses of mineral nutrients. During the recovery period, the plants were kept under ideal water supply.

### 4.4. Leaf Gas Exchange, Chlorophyll Fluorescence, and Relative Chlorophyll Index

Measurements of leaf gas exchange (net photosynthesis rate—*A*, transpiration rate—*E*, and stomatal conductance—*gs*) were performed on mature leaves of *C. prunifera* and *C. madagascariensis* seedlings at 0, 8, 12, 16, and 20 days of exposure to flooding stress and at 32 and 40 days (recovery periods), using a portable infrared gas analyzer (LC-Pro-SD, ADC Bioscientific Ltd., Hoddesdon, Hertfordshire, UK). Measurements were always taken in the morning, between 8:00 and 11:00 a.m., under ambient air temperature and relative humidity. The light intensity used in the leaf gas exchange measurements was 1600 µmol m^−2^ s^−1^.

The maximum efficiency of photosynthesis II, expressed by the Fv/Fm ratio, and the relative chlorophyll index were determined at 0, 8, 12, 16, and 20 days of exposure to flooding stress and at 32 and 40 days (recovery periods). Evaluations were performed using a portable fluorometer (Multi-mode Chlorophyl Fluorometer, model OS5p, Opti-Sciences, Hudson, NH, USA) and a portable chlorophyll meter (SPAD 502, Minolta Co., Ltd., Osaka, Japan), respectively. Measurements were taken at the same time and on the same leaves used to obtain leaf gas exchange data.

### 4.5. Morphological Responses and Biomass Production

At 0, 8, 12, 16, and 20 days of exposure to flooding stress, the height and stem diameter of the plants were measured using a metric tape measure and a digital caliper, respectively. Plant height was obtained by the vertical distance between the plant collar and the tip of the largest vertically stretched leaf, expressed in cm. Stem diameter was obtained at a height of approximately 3 cm from the soil surface, expressed in mm. Absolute growth rates in height (AGR-PH, cm day^−1^) and stem diameter (AGR-SD, mm day^−1^) were estimated according to the methodology described by [37].

The seedlings were collected 20 days after the last day of flooding stress, and the impacts of different flooding times were evaluated after a further 20-day recovery period. Leaf area was determined using an area integrator (LI-3100, Li-Cor, Inc., Lincoln, NE, USA). The root system was separated into three layers in each pot (0–8; 8–16; and 16–24 cm). All plant parts were placed in labeled paper bags and dried in a forced-air oven, maintaining a temperature between 65 and 70 °C. After drying, each sample was weighed on an analytical scale to measure the dry mass (shoots and roots), and the values were expressed in grams. Specific leaf area (SLA) was obtained by the ratio of total leaf area to dry leaf biomass, expressed in cm^2^ g^−1^.

### 4.6. Mineral Nutrient

For the evaluation of the nutritional status of the plants, lyophilized and ground leaf tissues were used. To determine the leaf concentrations of phosphorus, calcium, and magnesium, the plant material was subjected to wet digestion with nitric and perchloric acid, HNO_3_ + HClO_4_ in a 3:1 ratio [38]. Calcium and magnesium analyses were performed by atomic absorption spectrophotometry. Phosphorus was determined following the method proposed by [38]. Nitrogen content was obtained by sulfuric acid digestion extraction and determination by the Kjeldahl method. The extracts used for the determination of chloride, sodium, and potassium were obtained according to [39]. The chloride content in the leaves was obtained according to [40]. Chloride readings were performed on a Shimadzu^®^ UV-1650PC spectrophotometer. The determination of sodium and potassium levels was obtained by flame photometry.

### 4.7. Data Analysis

The data were subjected to the Shapiro–Wilk normality test as a prerequisite for analysis of variance using the F-test. Species were compared using Tukey’s test at a probability level of up to 5%, and the effects of flood stress periods were tested by regression using the statistical software SISVAR^®^, Lavras, MG, Brazil [41].

## 5. Conclusions

Our results show that the two species adopt contrasting functional strategies in response to flooding, reflecting differences in their morphophysiological characteristics and use of soil resources. While some attributes favor the maintenance of net carbon assimilation and functional stability under stress, others confer competitive advantages in environments with excess water in the soil. These distinctions highlight that the plants’ response depends both on immediate plasticity and on the genetic characteristics of each species, directly influencing their survival, performance, and competitiveness in ecosystems subject to long periods of flooding.

The invasive species showed high ability for soil resources acquisition, with higher values for leaf mineral nutrient concentration, net photosynthetic rate, growth rate, biomass production, leaf area, and specific leaf area, regardless of the water regime. Under flooding, the invasive species produced adventitious roots and accumulated a greater proportion of roots in the surface soil layer. In addition, the net photosynthetic rate in leaves was higher and less impacted by flooding in the invasive species, despite the relatively high sodium concentration in the mature leaves. On the other hand, the native species presented better root distribution in the soil, increased the root-to-shoot ratio, and was less impacted in terms of total biomass production.

The results indicate that the characteristics of *C. prunifera*, such as slow growth rate, low specific leaf area, and morphological adaptations of the root system, may ensure greater stability in net carbon assimilation in the whole plant under flooding. However, the high efficiency for mineral nutrients accumulation and rapid growth of the *C. madagascariensis* may impact the future renewal of carnauba palm groves in the Caatinga ecosystem, directly affecting the seedling establishment.

## Figures and Tables

**Table 1:** Root dry mass in soil layers of 0–8, 8–16 and 16–24 cm in *C. prunifera* and *C. madagascariensis* seedlings as a function of flooding stress periods. Values represent the mean ± standard error (n = 5).

	Soil Depth (cm)	Flooding Stress (Days)				
		0	8	12	16	20
		Roots Dry Mass (g per Plant)				
	0–8	1.51 ± 0.29	1.89 ± 0.26	2.01 ± 0.71	2.50 ± 0.17	2.36 ± 0.15
*C. prunifera*	8–16	1.83 ± 0.14	0.98 ± 0.13	0.59 ± 0.05	0.87 ± 0.22	0.87 ± 0.13
	16–24	1.01 ± 0.31	1.33 ± 0.26	1.62 ± 0.38	0.60 ± 0.13	0.73 ± 0.27
	0–8	6.76 ± 0.69	7.12 ± 0.57	7.15 ± 1.34	7.17 ± 0.47	8.11 ± 1.49
*C. madagascariensis*	8–16	4.96 ± 0.88	1.36 ± 0.22	1.90 ± 0.37	2.51 ± 0.87	1.42 ± 0.53
	16–24	6.58 ± 2.80	2.41 ± 0.76	1.90 ± 0.62	0.92 ± 0.32	0.86 ± 0.49

## Data Availability

The original contributions presented in this study are included in the article. Further inquiries can be directed to the corresponding author.

## References

[1] Essl F., Lenzner B., Bacher S., Bailey S., Capinha C., Daehler C., Roura-Pascual N. (2020). Drivers of future alien species impacts: An expert-based assessment. Glob. Change Biol..

[2] Barroso F.R.G., Seier M.K., Williamns F., Costa R.C., Araújo F.S., Mantovani W. (2024). Socioeconomic and environmental impacts in the carnaúba production chain by invasions of devil’s claw (*Cryptostegia madagascariensis*). Rev. Bras. Geogr. Fis..

[3] Miles L., Newton A.C., Defries R.S., Ravilious C., May I., Blyth S., Kapos V., Gordon J.E. (2006). A global overview of the conservation status of tropical dry forests. J. Biogeogr..

[4] Santos M.G., Oliveira M.T., Figueiredo K.V., Falcão H.M., Arruda E.C.P., Cortez J.Á., Sampaio E.V.S.B., Ometto J.P.H.B., Menezes R.S.C., Oliveira A.F.M. (2014). Caatinga, the Brazilian dry tropical forest: Can it tolerate climate changes?. Theor. Exp. Plant Physiol..

[5] Campos D.A., Andrade E.M., Castanho A.A., Feitosa R.C., Palácio H.Q.A. (2020). Biomass dynamics in a fragment of Brazilian tropical forest (Caatinga) over consecutive dry years. Appl. Sci..

[6] Costa M.S., Ferreira K.E.B., Botosso P.C., Callado C.H. (2015). Growth analysis of five Leguminosae native tree species from a seasonal semidecidual lowland forest in Brazil. Dendrochronologia.

[7] Silva E.B., Kavamura V.N., Freitas F.M.M., Souza A.J., Pereira A.P.A. (2025). Current knowledge of carnauba plant (*Copernicia prunifera*): Current stage, trends, and future perspectives. Environments.

[8] Arruda G.M.T., Calbo M.E.R. (2004). Efeitos da inundação no crescimento, trocas gasosas e porosidade radicular da carnaúba (*Copernicia prunifera*). Acta Bot. Bras..

[9] Sousa F.Q., Andrade L.A., Xavier K.R.F. (2016). *Cryptostegia madagascariensis* Bojer ex Decne.: Impacts on natural regeneration in Caatinga fragments. Rev. Bras. Cienc. Agrar..

[10] Barbosa E.M., Bonilla O.H., Lucena E.M.P., Andrade L.M. (2019). Estrutura de um fragmento de Caatinga infestado por *Cryptostegia madagascariensis* Bojer ex Decne. Rev. Bras. Geogr. Fis..

[11] Dillenburg L.R., Teramura A.H., Forseth I.N., Whigham D.F. (1995). Photosynthetic and biomass allocation responses of *Liquidambar styraciflua* (Hamamelidaceae) to vine competition. Am. J. Bot..

[12] Toledo-Aceves T., Swaine M.D. (2008). Above- and below-ground competition between the liana *Acacia kamerunensis* and tree seedlings in contrasting light environments. Plant Ecol..

[13] Medeiros W.J.F., Lacerda C.F., Zandavalli R.B., Araújo I.C.S., Sousa C.H.C., Bezerra A.M.E., Ribeiro A.A., Braz R.S. (2023). The ecophysiological responses of *Copernicia prunifera* palm trees to soil constraints and competition with invasive *Cryptostegia madagascariensis* in tropical dryland. Acta Physiol. Plant..

[14] Medeiros W.J.F., Oliveira F.Í.F., Lacerda C.F., Sousa C.H.C., Cavalcante L.F., Silva A.R.A., Ferreira J.F.S. (2018). Isolated and combined effects of soil salinity and waterlogging in seedlings of ‘Green Dwarf’ coconut. Semin. Ciênc. Agrar..

[15] Yu X., Luo N., Yan J., Tang J., Liu S., Jiang Y. (2012). Differential growth response and carbohydrate metabolism of global collection of perennial ryegrass accessions to submergence and recovery following de-submergence. J. Plant Physiol..

[16] Singh A. (2015). Soil salinization and waterlogging: A threat to environment and agricultural sustainability. Ecol. Indic..

[17] Ren B., Zhang J., Dong S., Liu P., Zhao B. (2016). Effects of waterlogging on leaf mesophyll cell ultrastructure and photosynthetic characteristics of summer maize. PLoS ONE.

[18] Armstrong W., Beckett P.M., Colmer T.D., Setter T.L., Greenway H. (2019). Tolerance of roots to low oxygen: ‘anoxic’ cores, the phytoglobin–nitric oxide cycle, and energy or oxygen sensing. J. Plant Physiol..

[19] Silva C.J., Amarante L. (2020). Short-term nitrate supply decreases fermentation and oxidative stress caused by waterlogging in soybean plants. Environ. Exp. Bot..

[20] Liu M., Jiang Y. (2015). Genotypic variation in growth and metabolic responses of perennial ryegrass exposed to short-term waterlogging and submergence stress. Plant Physiol. Biochem..

[21] Pompeniano A., Reyes T.H., Moles T.M., Scartazza A.R. (2019). Photosynthetic and growth responses of *Arundo donax* L. plantlets under different oxygen deficiency stresses and reoxygenation. Front. Plant Sci..

[22] García I., Mendonza R. (2014). *Lotus tenuis* seedlings subjected to drought or waterlogging in a saline sodic soil. Environ. Exp. Bot..

[23] Andrade L.A. (2013). Plantas Invasoras: Espécies Vegetais Exóticas Invasoras da Caatinga e Ecossistemas Associados.

[24] Moro M.F., Macedo M.B., Moura-Fé M.M., Castro A.S.F., Costa R.C. (2015). Vegetação, unidades fitoecológicas e diversidade paisagística do estado do Ceará. Rodriguésia.

[25] Ferreira C.D.S., Piedade M.T.F., Tiné M.A.S., Rossatto D.R., Parolin P., Buckeridge M.S. (2009). The role of carbohydrates in seed germination and seedling establishment of *Himatanthus sucuuba*, an Amazonian tree with populations adapted to flooded and non-flooded conditions. Ann. Bot..

[26] Jackson M.B., Armstrong W. (1999). Formation of aerenchyma and the processes of plant ventilation in relation to soil flooding and submergence. Plant Biol..

[27] Jackson M.B., Colmer T.D. (2005). Response and adaptation by plants to flooding stress. Ann. Bot..

[28] Taiz L., Zeiger E., Moller I.M., Murphy A. (2021). Fundamentos de Fisiologia Vegetal.

[29] Schmidt F., Fortes M.Á., Wesz J., Buss G.L., Sousa R.O. (2013). Impacto do manejo da água na toxidez por ferro no arroz irrigado por alagamento. Rev. Bras. Ciênc. Solo.

[30] Nawaz T., Hameed M., Ashraf M., Ahmad M.S.A., Batool R., Fatima S. (2014). Anatomical and physiological adaptations in aquatic ecotypes of *Cyperus alopecuroides* Rottb. under saline and waterlogged conditions. Aquat. Bot..

[31] Huang D., Wang D., Ren Y. (2019). Using leaf nutrient stoichiometry as an indicator of flood tolerance and eutrophication in the riparian zone of the Lijang River. Ecol. Indic..

[32] Milroy S.P., Bange M.P., Thongbai P. (2009). Cotton leaf nutrient concentrations in response to waterlogging under field conditions. Field Crops Res..

[33] Zhang Y., Liu G., Dong H., Li C. (2021). Waterlogging stress in cotton: Damage, adaptability, alleviation strategies, and mechanisms. Crop J..

[34] Liao C.T., Lin C.H. (2001). Physiological adaptation of crop plants to flooding stress. Proc. Natl. Sci. Counc..

[35] Zhang Y., Chen X., Geng S., Zhang X. (2025). A review of soil waterlogging impacts, mechanisms, and adaptive strategies. Front. Plant Sci..

[36] Mohamed R., Khalil W., Zaghloul M. (2023). Exploring the physiological and molecular mechanisms of halophytes’ adaptation to high salinity environments: Implications for enhancing plant salinity tolerance. Catrina Int. J. Environ. Sci..

[37] Benincasa M.M.P. (2003). Análise de Crescimento de Plantas: Noções Básicas.

[38] EMBRAPA (2009). Manual de Análises Químicas de Solos, Plantas e Fertilizantes.

[39] Cataldo D.A., Maroon M., Schrader L.E., Youngs V.L. (1975). Rapid colorimetric determination of nitrate in plant tissue by nitration of salicylic acid. Commun. Soil Sci. Plant Anal..

[40] Gaines T.P., Parker M.B., Gascho G.J. (1984). Automated determination of chlorides in soil and plant tissue by sodium nitrate. Agron. J..

[41] Ferreira D.F. (2010). SISVAR^®^: Sistema de Análise de Variância para Dados Balanceados.

